# The Prognostic Value of the Prognostic Nutritional Index in Operable High-Grade Glioma Patients and the Establishment of a Nomogram

**DOI:** 10.3389/fonc.2021.724769

**Published:** 2022-01-14

**Authors:** Qian He, Wei Zhao, Qinglan Ren

**Affiliations:** ^1^ Department of Oncology, Affiliated Dongguan People’s Hospital, Southern Medical University, Dongguan, China; ^2^ Department of Oncology, The Second Affiliated Hospital of Chongqing Medical University, Chongqing, China; ^3^ Department of Oncology, The First Affiliated Hospital of Chongqing Medical University, Chongqing, China

**Keywords:** prognostic nutritional index, high-grade glioma, prognostic, overall survival, nomogram

## Abstract

**Background:**

Studies confirmed the predictive value of the prognostic nutrition index (PNI) in many malignant tumors. However, it did not reach a consensus in glioma. Therefore, this study investigated the prognostic value of preoperative PNI in operable high-grade glioma and established a nomogram.

**Methods:**

Clinical data of high-grade glioma patients were retrospectively analyzed. The primary endpoint was overall survival (OS). Survival analysis was conducted by the Kaplan–Meier method, log-rank test, and Cox regression analysis. A nomogram was established. The prediction effect of the nomogram covering PNI was verified by area under the curve (AUC).

**Results:**

A total of 91 operable high-grade glioma patients were included. Kaplan–Meier analysis showed that among grade IV gliomas (n = 55), patients with higher PNI (>44) showed a trend of OS benefit (p = 0.138). In grade III glioma (n = 36), patients with higher PNI (>47) had longer OS (p = 0.023). However, the intersecting Kaplan–Meier curve suggested that there may be some confounding factors. Cox regression analysis showed that higher PNI was an independent prognostic factor for grade IV glioma (HR = 0.388, p = 0.040). In grade III glioma, there was no statistically relationship between PNI levels and prognosis. When evaluating the prognostic ability of PNI alone by ROC, the AUC in grade III and IV gliomas was low, indicating that PNI alone had poor predictive power for OS. Interestingly, we found that the nomogram including preoperative PNI, age, extent of resection, number of gliomas, and MGMT methylation status could predict the prognosis of patients with grade IV glioma well.

**Conclusion:**

The PNI level before surgery was an independent prognostic factor for patients with grade IV glioma. The nomogram covering PNI in patients with grade IV glioma also proved the value of PNI. However, the value of PNI in grade III glioma needs to be further evaluated. More prospective studies are needed to verify this conclusion.

## Introduction

The overall prognosis of patients with high-grade glioma is poor, and the survival of patients varied greatly, and their 5-year survival rate fluctuates between 5.5% and 75.2% (1). Therefore, it is very important to accurately predict the prognosis of patients. Research showed that the prognosis of patients with high-grade glioma was related to factors such as patient age, tumor characteristics, and treatment methods (1). Nevertheless, the accuracy in predicting the prognosis of high-grade gliomas is still limited. It is necessary to find more prognostic factors to comprehensively evaluate the prognosis of patients with high-grade glioma.

Studies showed that the nutritional status and inflammation of patients may affect the antitumor effect (2–5). The prognostic nutritional index (PNI) is based on the serum albumin count and lymphocyte count and is a comprehensive index for evaluating the nutritional status and inflammatory state of the body. Its calculation formula is serum albumin concentration (g/L) + 5× total lymphocyte count (10^9^/l). More and more studies found that higher PNI can be used for independent factors for better prognosis of various malignant tumors, such as lung cancer (6), esophageal cancer (7), breast cancer (8), nasopharyngeal cancer (9), colorectal cancer (10), gastric cancer (11), and biliary tract cancer (12). However, there is no consensus on the role of PNI in glioma (13). Therefore, this study intended to explore the prognostic value of PNI in patients with operable high-grade glioma.

## Methods

### Patients

The clinical data of operable high-grade glioma patients from December 2013 to December 2019 in the First Affiliated Hospital of Chongqing Medical University were analyzed retrospectively. The inclusion criteria include the following (1): patients underwent tumor resection (2); patients with high-grade glioma diagnosed according to the 2016 WHO classification and global standard classification (3); patients who completed the “STUPP” radiotherapy and chemotherapy protocol (4); regular follow-up; and (5) blood routine and serum albumin examination within 1 week before operation. The exclusion criteria include the following (1): incomplete clinical data (2); receiving other treatments before tumor resection; and (3) having infection or inflammatory disease during the last month.

### Data Collection and Hematological Examination

Clinical data of patients were collected including age, gender, tumor grade, histological type, tumor site, tumor number, and extent of resection. The extent of surgical resection was determined according to the preoperative and postoperative MRI and the surgeon’s intraoperative judgment. Postoperative MRI was obtained within 24–72 h after surgery. We used Carestream Vue PACS software to calculate the percentage of resection based on the preoperative MRI and postoperative MRI within 24–72 h and marked the tumor extent according to T1-enhanced sequence images. The percentage of tumor resection = (preoperative tumor volume-residual tumor volume)/preoperative tumor volume * 100%. No tumor remaining was considered as gross total resection (GTR), tumor resection extent > 90% was considered as near total resection (NTR), tumor resection extent between 80% and 90% was considered as subtotal resection (STR), and tumor resection extent <80% was considered as partial resection (PR). Blood routine and serum albumin results before surgery were collected. The cutoff value of the optimal PNI was obtained by X-Tile software.

### Follow-Up

The primary endpoint of the study was OS. OS was defined as the time from the day of surgery to the death of the patient or the final follow-up. There were 91 patients who met the inclusion and exclusion criteria and were successfully followed up. The follow-up ended on September 12, 2020.

### Statistical Analysis

We used the independent sample T test or Mann–Whitney U test to compare continuous variables, and the chi-square test or Fisher’s exact probability test to compare categorical variables. The Kaplan–Meier method, log-rank method, and Cox regression model were used for survival analysis. Survival was analyzed by calculating the hazard ratio (HR) and 95% confidence interval (CI). The prognostic ability of PNI was evaluated by the area under the curve (AUC) through the ROC curve. A nomogram covering the PNI was built, and the C index was calculated. The predictive effect of the nomogram was verified by the calibration curve through bootstrap sampling 1,000 times. The predictive ability of the nomogram covering PNI and the predictive model without PNI was compared by the ROC curve. SPSS version 25, X-Tile version 3.6.1, and R version 4.0.2 were used for data analysis. The statistical significance of the p value was set at 0.05 (double-sided).

## Results

### Patient Characteristics

A total of 91 high-grade glioma patients (36 cases of grade III and 55 cases of grade IV) were enrolled. The median age was 50 years (range 18–79 years). **Table 1** showed the characteristics of grade III and grade IV glioma patients and their correlation with preoperative PNI. In patients with grade III and grade IV glioma, the best cutoff values of preoperative PNI were 47 and 44, respectively.

**Table 1:** Baseline patient characteristics stratified by preoperative PNI in grade III glioma and grade IV glioma.

	Grade III glioma					Grade IV glioma				
Variables	Total(n = 36)		PNI ≤47(n = 9, 25%)	PNI >47(n = 27, 75%)	p	Total(n = 55)		PNI ≤44(n = 7, 12.7%)	PNI >44(n = 48, 87.3%)	p
	n/mean ± SD	%	n/mean ± SD	n/mean ± SD		n/mean ± SD	%	n/mean ± SD	n/mean ± SD	
**Age**	48.4 ± 10.30		53.2 ± 10.95	46.7 ± 9.75	0.103	51.1 ± 14.26		51.7 ± 16.59	51.0 ± 14.08	0.906
**Sex**										
Female	14	38.9	3	11	1.000	29	52.7	4	25	1.000
Male	22	61.1	6	16		26	47.3	3	23	
**Histology**										
AA	21	58.3	5	16	0.681	NA				
AO	13	36.1	3	10		NA				
GBM	NA					55	100	7	48	NA
NOS	2	5.6	1	1		NA				
**Main location**										
Frontal	25	69.4	5	20	0.531	32	58.2	5	27	0.686
Parietal	4	11.1	1	3	1.000	18	32.7	2	16	1.000
Occipital	2	5.6	1	1	0.443	9	16.4	0	9	0.480
Temporal	9	25.0	3	6	0.824	12	21.8	2	10	1.000
Insular	1	2.8	1	0	0.250	2	3.6	0	2	1.000
**No. of glioma**										
Single	36	100	9	27	NA	49	89.1	6	43	0.577
Multiple	0	0.0	0	0		6	10.9	1	5	
**Extent of resection**										
PR	2	5.6	0	2	1.000	0	0.0	0	0	0.513
STR	3	8.3	1	2		6	10.9	0	6	
NTR	6	16.7	1	5		8	14.5	0	8	
GTR	25	69.4	7	18		41	74.5	7	34	
**IDH mutation**										
No	2	5.6	0	2	1.000	46	83.6	6	40	1.000
Yes	34	94.4	9	25		9	16.4	1	8	
**MGMT methylation**										
No	14	38.9	5	9	0.430	34	61.8	4	30	1.000
Yes	22	61.1	4	18		21	38.2	3	18	
**1p19q deletion**										
No	23	63.9	6	17	1.000	0	0	0	0	NA
Yes	13	36.1	3	10		0	0	0	0	
**Ki-67**	16.3 ± 14.44		22.2 ± 21.38	14.3 ± 11.12	0.311	27.6 ± 14.43		35.0 ± 18.03	26.5 ± 13.72	0.145
**Epilepsy before surgery**										
No	23	63.9	7	16	0.548	45	81.8	6	39	1.000
Yes	13	36.1	2	11		10	18.2	1	9	

AA, anaplastic astrocytomas; AO, anaplastic oligodendrogliomas; GBM, glioblastoma; GTR, gross total resection; n, number; NA, not applicable; No., number; NOS, not otherwise specified; NTR, near total resection; PNI, prognostic nutritional index; PR, partial resection; SD, Std. deviation; STR, subtotal resection.

### Survival Analysis

The median follow-up time for all patients was 21 months (95% CI: 23.4–31.4 months). The median OS for grade III glioma was 31 months (95% CI: 28.9–41.6 months), and the median OS for grade IV glioma was 17 months (95% CI: 17.5–27.1 months). Among patients with grade III glioma, the median OS of patients in the preoperative low PNI (≤47) group and high PNI (>47) group was 22 and 37 months, respectively. In grade IV glioma, the median OS for the preoperative lower PNI (≤44) group and higher PNI (>44) group was 14 and 17.5 months.

**Figure 1:**
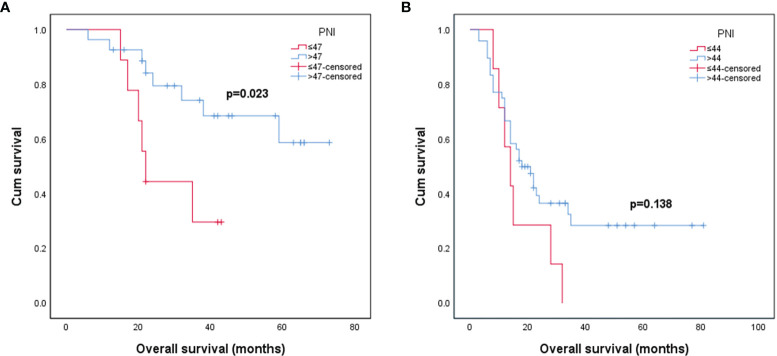
Kaplan–Meier survival curves for patients stratified based on preoperative PNI in patients with grade III glioma **(A)** and patients with grade IV glioma **(B)**. (PNI, prognostic nutritional index).

The Kaplan–Meier survival curve and log-rank test were used to analyze the prognosis of patients in different PNI states. It was found that in grade III glioma, the OS of patients with preoperative high PNI status was significantly longer than that of preoperative low PNI status (**Figure 1A**, p = 0.023). However, the Kaplan–Meier curve intersected, suggesting that in grade III gliomas, there may be confounding factors that make patients with high PNI have longer OS, so we conducted a multivariate analysis. Grade IV glioma patients with higher preoperative PNI also showed a similar trend of obtain better prognosis (**Figure 1B**, p = 0.138).

Cox regression analysis showed that in grade IV glioma, preoperative PNI level, extent of resection, and MGMT methylation status were significantly correlated with OS (**Table 2**). Since primary and secondary grade IV gliomas were usually distinguished according to the wild type and mutant type of IDH (14), and the predictive value of preoperative PNI in patients with different types of grade IV gliomas may be different, we performed subgroup analysis on IDH wild-type grade IV glioma patients. The results showed that patients with higher preoperative PNI had a tendency to obtain OS benefits (HR = 0.476, p = 0.126, **Table 3**). We did not further analyze the role of PNI in secondary grade IV gliomas due to the small number of IDH mutated grade IV gliomas patients included (n = 9). In grade III glioma, age, histological type, extent of resection, MGMT methylation status, and Ki-67 level were independent prognostic factors that affect OS in patients (**Table 4**), and patients with higher preoperative PNI showed a better prognosis only in the univariate analysis (HR = 0.301, p = 0.032).

**Table 2:** Univariate and multivariate Cox regression analyses for overall survival in grade IV glioma patients.

		Univariate Cox regression			Multivariate Cox regression		
Variable		p value	HR	(95% CI)	p value	HR	(95% CI)
Age		0.105	1.019	(0.996 -1.042)	0.382	1.010	(0.988 -1.032)
Sex	Female	ref					
	Male	0.537	1.222	(0.646 -2.313)			
No. of glioma	Single	ref			ref		
	Multiple	0.016*^a^*	3.321	(1.247 -8.847)	0.356	1.647	(0.571 -4.755)
Extent of resection	STR+PR	ref					
	GTR+NTR	0.001*^a^*	0.200	(0.078 -0.514)	0.002*^a^*	0.201	(0.073 -0.551)
IDH mutation	No	ref					
	Yes	0.004*^a^*	0.171	(0.051 -0.574)	0.096	0.320	(0.084 -1.223)
MGMT methylation	No	ref					
	Yes	0.001^a^	0.293	(0.14 -0.615)	0.036*^a^*	0.408	(0.177 -0.941)
Ki-67		0.580	1.006	(0.985 -1.028)	0.880	1.002	(0.977 -1.028)
Epilepsy before surgery	No	ref					
	Yes	0.197	0.528	(0.200 -1.392)			
PNI	≤44	ref			ref		
	>44	0.155	0.548	(0.239 -1.255)	0.040*^a^*	0.388	(0.158 -0.956)

CI, confidence interval; GTR, gross total resection; HR, hazard ratio; No., number; NTR, near total resection; PNI, prognostic nutritional index; PR, partial resection; ref, reference; STR, subtotal resection. ^a^Statistically significant (p < 0.05).

**Table 3:** Univariate and multivariate Cox regression analyses for overall survival in the IDH wide-type IV glioma subgroup.

		Univariate Cox regression			Multivariate Cox regression		
Variable		p value	HR	(95% CI)	p value	HR	(95% CI)
Age		0.513	1.007	(0.985 1.030)	0.834	1.002	(0.980 -1.025)
Sex	Female	ref					
	Male	0.767	1.106	(0.568 -2.152)			
No. of glioma	Single	ref			ref		
	Multiple	0.058	2.568	(0.967 -6.822)	0.731	1.246	(0.356 -4.365)
Extent of resection	STR+PR	ref			ref		
	GTR+NTR	0.004*^a^*	0.254	(0.099 -0.652)	0.005*^a^*	0.234	(0.085 -0.645)
MGMT methylation	No	ref			ref		
	Yes	0.087	0.504	(0.230 -1.105)	0.087	0.479	(0.207 -1.112)
Ki-67		0.914	0.999	(0.977 -1.021)			
Epilepsy before surgery	No	ref			ref		
	Yes	0.012*^a^*	3.684	(1.338 -10.143)	0.246	2.129	(0.594 -7.626)
PNI	≤44	ref			ref		
	>44	0.369	0.666	(0.274 -1.617)	0.126	0.476	(0.184 -1.232)

CI, confidence interval; GTR, gross total resection; HR, hazard ratio; No., number; NTR, near total resection; PNI, prognostic nutritional index; PR, partial resection; ref, reference; STR, subtotal resection. ^a^Statistically significant (p < 0.05).

**Table 4:** Univariate and multivariate Cox regression analyses for overall survival in grade III glioma patients.

		Univariate Cox regression			Multivariate Cox regression		
Variable		p value	HR	(95% CI)	p value	HR	(95% CI)
Age		0.008*^a^*	1.078	(1.020 -1.139)	0.002*^a^*	1.137	(1.047 -1.234)
Sex	Female	ref					
	Male	0.914	1.062	(0.355 -3.181)			
Histology	AA	ref			ref		
	AO	0.525	0.682	(0.210 -2.220)	0.028*^a^*	0.114	(0.016 -0.789)
	NOS	0.875	1.183	(0.147 -9.483)	0.093	0.017	(0.000 -1.977)
Extent of resection	STR+PR	ref			ref		
	GTR+NTR	0.004*^a^*	0.195	(0.063 -0.599)	0.041*^a^*	0.237	(0.060 -0.941)
IDH mutation	No	ref			ref		
	Yes	0.848	0.819	(0.106 -6.311)	0.646	0.574	(0.053 -6.157)
MGMT methylation	No	ref			ref		
	Yes	0.187	0.487	(0.167 -1.418)	0.016*^a^*	0.128	(0.024 -0.687)
1p19q deletion	No	ref					
	Yes	0.503	0.672	(0.210 -2.149)			
Ki-67		0.018*^a^*	1.043	(1.007 -1.080)	0.037*^a^*	1.071	(1.004 -1.143)
Epilepsy before surgery	No	ref					
	Yes	0.081	0.318	(0.088 -1.151)			
PNI	≤47	ref			ref		
	>47	0.032*^a^*	0.301	(0.100 -0.904)	0.974	1.024	(0.239 -4.390)

AA, anaplastic astrocytomas; AO, anaplastic oligodendrogliomas; CI, confidence interval; GTR, gross total resection; HR, hazard ratio; NOS, not otherwise specified; NTR, near total resection; PNI, prognostic nutritional index; PR, partial resection; ref, reference; STR, subtotal resection.^a^Statistically significant (p lt 0.05).

**Figure 2:**
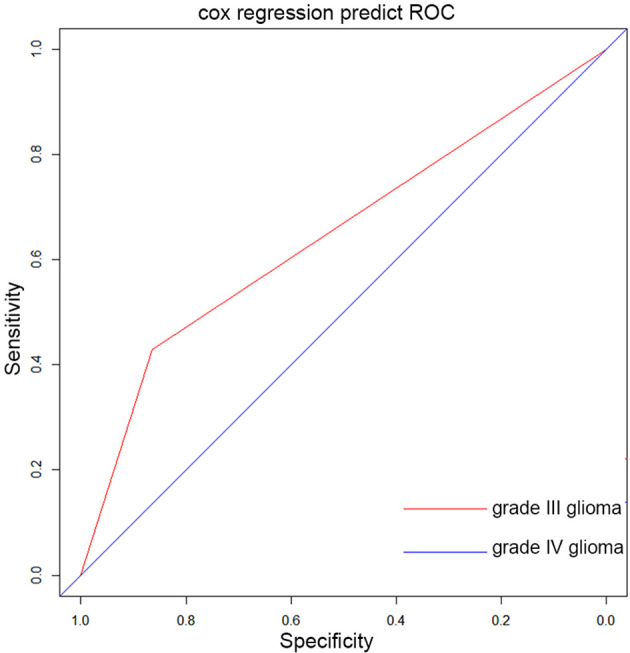
The ROC curve of PNI for grade III and grade IV glioma patients.

When assessing the prognostic ability of PNI in grade III and IV gliomas through the ROC curve, we found that the AUC was not high (0.65 and 0.50, respectively, **Figure 2**), which indicated that PNI alone had a limited effect of predicting OS in patients with high-grade glioma.

### The Establishment of a Nomogram

**Figure 3:**
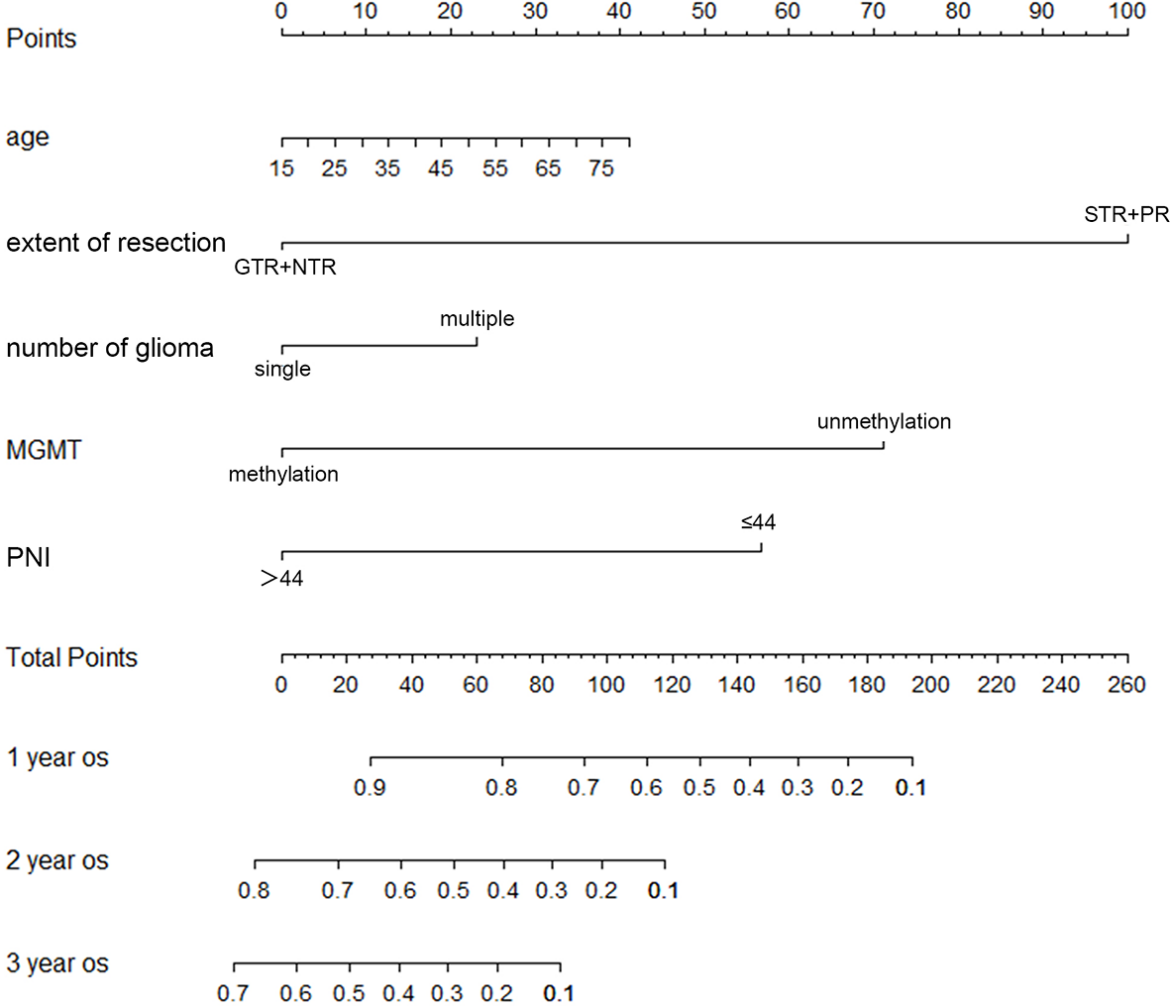
The nomogram for survival of patients with grade IV glioma. (GTR, gross total resection; NTR, near total resection; PNI, prognostic nutritional index; PR, partial resection; STR, subtotal resection).

**Figure 4:**
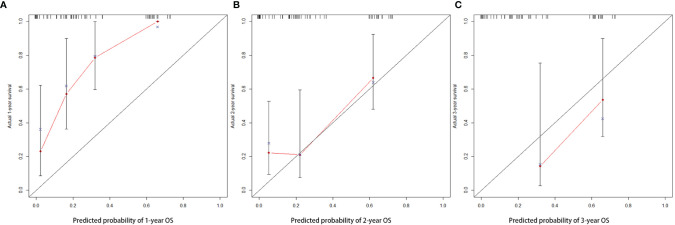
The calibration curve of nomogram in patients with grade IV glioma at 1 **(A)**, 2 **(B)**, and 3 years **(C)**.

**Figure 5:**
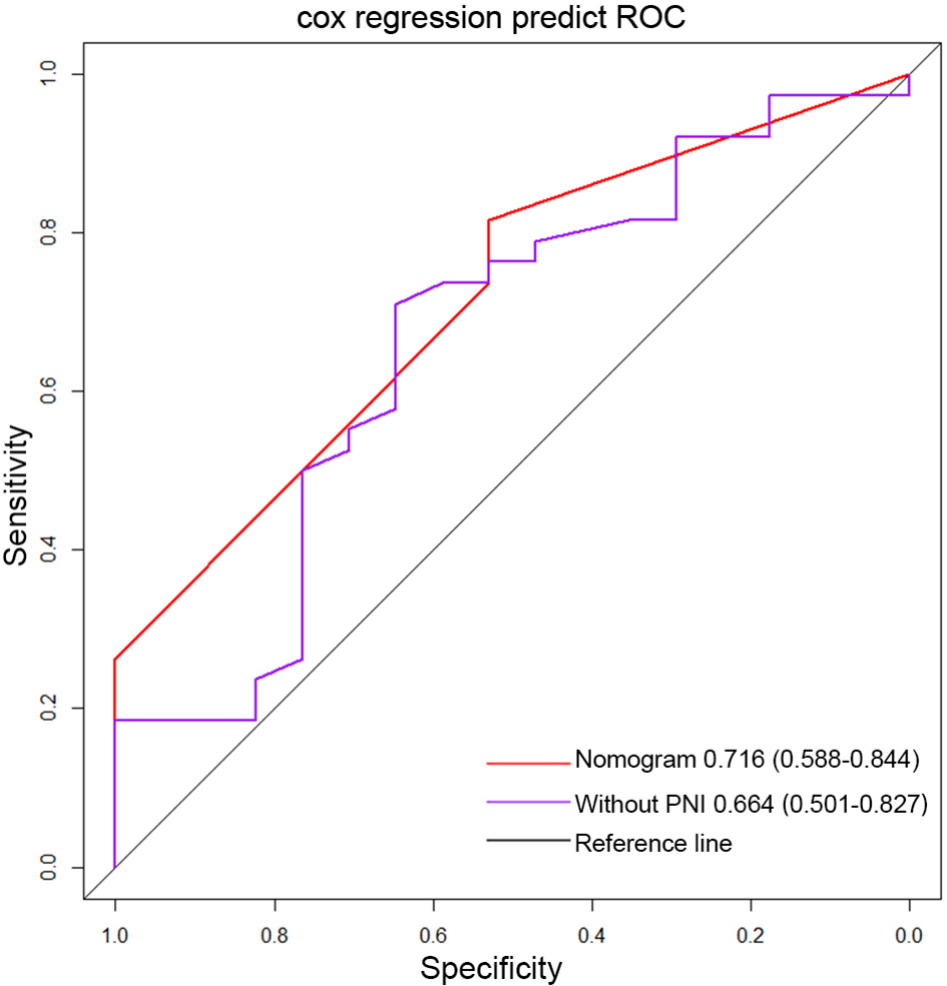
The ROC curve of the nomogram covering PNI and the ROC curve of traditional markers in grade IV glioma. (PNI, prognostic nutritional index).

Important factors associated with the prognosis of high-grade gliomas included age, extent of resection, number of gliomas, and MGMT methylation status. Since our study showed that preoperative PNI level was an independent prognostic factor in grade IV glioma patients, however, in grade III glioma, a significant correlation between PNI and OS was only shown in univariate analysis, and the number of patients with grade III glioma included was relatively small. Therefore, we only drew a nomogram in grade IV gliomas covering preoperative PNI, age, extent of resection, number of gliomas, and MGMT methylation status (**Figure 3**) and evaluated the predictive effect of the nomogram. The C-index of the nomogram was 0.771 (95% CI: 0.708–0.834). The calibration curves for the 1-, 2-, and 3-year survival of the patients showed that when predicting the 2-year survival rate, the agreement between the prediction and the observation results was the best (**Figures 4A–C**), indicating that the repeatability of the nomogram was reliable. ROC analysis showed that the AUC of the nomogram covering the preoperative PNI was higher than the AUC of the prediction model without PNI (AUC = 0.716, 95% CI: 0.588–0.844 and AUC = 0.664, 95% CI: 0.501–0.827, respectively; **Figure 5**). This suggested that the nomogram could more accurately predict the prognosis of patients with grade IV glioma.

## Discussion

The nutritional status, inflammatory status, and immune function of patients with malignant tumors were often considered to be related to the prognosis (5, 15). Serum albumin levels were often used to assess the nutritional status of patients, and lymphocytes were often used to assess the immune function and inflammatory status of patients (16, 17). Many studies proved the prognostic value of serum albumin and lymphocytes in malignant tumors: lower albumin levels were linked to poor prognosis for cancer patients (13, 18), and high levels of lymphocytes may indicate a better prognosis (19–21). As an easily accessible index that could comprehensively evaluate the nutritional status and inflammatory status of patients, more and more studies confirmed its prognostic value in a variety of malignant tumors (9, 11, 22, 23). However, the prognostic value of PNI in glioma has not yet reached consensus (13, 24, 25).

This study mainly explored the predictive value of preoperative PNI in patients with operable high-grade glioma. We only included patients with high-grade glioma who received standardized postoperative adjuvant radiotherapy and chemotherapy. After excluding the influence of treatment factors, it could better reflect the predictive ability of preoperative PNI. The IDH mutation frequency (94% and 16%, respectively) and MGMT methylation frequency (61% and 38%, respectively) of grade III and grade IV glioma reported in our study were consistent with the frequency reported in the past (26, 27). Some factors that affect the prognosis of patients with high-grade glioma, such as age, histological type, single or multiple gliomas, extent of resection, MGMT methylation status, and Ki-67 level, were verified in our results (27–32). However, the OS of patients with different IDH statuses did not show significant statistical differences. It may be related to the small number of IDH wild-type grade III glioma patients and IDH mutant grade IV glioma patients we included. Regarding PNI, consistent with most studies, we found that preoperative PNI showed a predictive value (13). Our study found that in grade III and grade IV gliomas, the median OS of patients with higher preoperative PNI was longer than that of patients with lower PNI. Preoperative PNI was an independent prognostic factor for grade IV glioma. However, in univariate analysis, patients with higher preoperative PNI only had trends to get better OS. This may be because OS was affected by other factors in addition to preoperative PNI. When a multivariate analysis was performed, the significance of preoperative PNI becomes apparent. Furthermore, through ROC analysis, the nomogram covering PNI also showed a better prognostic value compared with the prediction model without PNI. In the IDH wild-type grade IV glioma subgroup, higher PNI only showed a trend for better OS. This may be due to the small number of IDH wild-type IV glioma patients included (n = 46). In this study, PNI did not show a good prognostic effect in grade III gliomas. Although our study showed that PNI in grade III gliomas was statistically significant in univariate Cox regression and Kaplan–Meier analysis, the intersecting Kaplan–Meier curves suggested that there might be confounding factors. Multivariate Cox analysis showed that the PNI was not an independent prognostic factor for grade III gliomas. This may be due to the strong heterogeneity of grade III gliomas, and a single indicator may not be used as a reliable independent prognostic factor. Moreover, the number of grade III gliomas patients we included was small (n = 36), which may be insufficient to support the inclusion of more indicators for multivariate analysis. In the future, we will follow more patients to evaluate the predictive value of PNI in grade III gliomas. In addition, we included 2 IDH wild-type grade III glioma patients. Some IDH wild-type grade III glioma patients may be classified as grade IV glioma with the development of molecular pathology (33). In the future, we would include more patients and evaluate the prognostic value of PNI in different subtypes of high-grade gliomas. Although, through the ROC curve, we found that in high-grade glioma, the predictive effect of PNI alone was limited, the nomogram based on PNI showed good predictive performance. The AUC of the nomogram covering PNI was higher than that of the prediction model without PNI. This showed that the nomogram covering preoperative PNI was better than traditional biomarkers. The molecular pathological diagnosis of patients may change with the advancement of molecular detection in the future. We will continue to include more patients and further optimize the nomogram to more accurately predict the OS of patients with high-grade glioma.

As a cheap and convenient indicator, PNI may affect the prognosis of patients with malignant tumors in many ways. Many studies found that the level of albumin was related to the nutritional status of patients and the immune system (34). Lower serum albumin levels indicated an increased nutritional risk for malignant tumor patients. In addition, lower albumin levels could also interfere with the body’s immune response and reduce antitumor effects (34, 35). Lymphocytes were an important cell group for body immunity and an important factor for regulating immunity. Lymphocytes could limit tumor growth and metastasis through cytotoxicity and improve patient prognosis (36, 37). Therefore, high levels of PNI may often be associated with better prognosis in patients with malignant tumors. However, in high-grade gliomas, this conclusion was not always valid, and more clinical data were still needed to explore the prognostic value of PNI in high-grade gliomas. Due to the special microenvironment of glioma, its immune response was different from other solid tumors. First, most immune cells could not pass the blood–brain barrier smoothly, and high-grade gliomas were relatively “cold” tumors (38). Secondly, the ratio of CD4+ T cells, CD8+ T cells, regulatory T cells (Treg), and other types of T lymphocytes in the glioma microenvironment was different. Studies showed that the number of CD4+ T cells, CD8+ T cells, and Treg cells could affect the tumor immune response in gliomas (39–41). In addition, gliomas could also be divided into different subtypes according to the Tumor Genomics Atlas Project (TCGA), and the prognosis of different subtypes may be different (42, 43). These may be the reasons why the findings found in other malignant tumors did not necessarily exist in high-grade gliomas.

There was no doubt that this study had certain limitations (1). As a single-center, retrospective study, the number of patients included in this study was limited and the follow-up time was insufficient. Moreover, the number of people in the high-PNI and low-PNI groups was uneven. We will continue to follow up more patients to obtain more accurate results (2). The biomarker status of the tumor may affect the prognosis of patients with high-grade glioma. We would continue to follow up and include more patients with more accurate tumor molecular classification to verify our findings and optimize the nomogram (3). Patients with high-grade glioma may receive different treatments after surgery, such as tumor-treating fields and bevacizumab, and the duration of treatment may also be different. All of these may lead to bias in research results. More prospective clinical trials were needed to evaluate the prognostic value of PNI in patients with high-grade glioma (4). Although the OS predicted by the nomogram was in good agreement with the observed OS, the general applicability of the findings still needed to be confirmed by external verification.

## Conclusions

Preoperative PNI level was an independent prognostic factor for patients with grade IV glioma. The nomogram covering PNI could predict the prognosis of patients with grade IV glioma more accurately than traditional markers. The value of PNI in grade III glioma needs to be further evaluated. More large-scale prospective clinical trials are needed to evaluate the prognostic value of preoperative PNI in high-grade gliomas.

## Data Availability Statement

The original contributions presented in the study are included in the article/**Supplementary Material**. Further inquiries can be directed to the corresponding author.

## Ethics Statement

Ethical review and approval were not required for the study on human participants in accordance with the local legislation and institutional requirements. Written informed consent for participation was not required for this study in accordance with the national legislation and the institutional requirements.

## Author Contributions

(I) Conception and design: all authors. (II) Administrative support: none. (III) Provision of study materials or patients: QH, QR. (IV) Collection and assembly of data: QH, WZ. (V) Data analysis and interpretation: QH, QR. (VI) Manuscript writing: all authors. (VII) Final approval of manuscript: all authors. All authors contributed to the article and approved the submitted version.

## Conflict of Interest

The authors declare that the research was conducted in the absence of any commercial or financial relationships that could be construed as a potential conflict of interest.

## Publisher’s Note

All claims expressed in this article are solely those of the authors and do not necessarily represent those of their affiliated organizations, or those of the publisher, the editors and the reviewers. Any product that may be evaluated in this article, or claim that may be made by its manufacturer, is not guaranteed or endorsed by the publisher.
