# Supplementary material for: The Prognostic Value of the Prognostic Nutritional Index in Operable High-Grade Glioma Patients and the Establishment of a Nomogram
Source: Front Oncol. 2022 Jan 14;11:724769. doi: 10.3389/fonc.2021.724769 (PMC8795507; doi:10.3389/fonc.2021.724769)
Supplement: Supplementary file 1 [file DataSheet_1.doc]

| Data S1. Original data. | | | | | | | | | | | | | | | | | | | |
| --- | --- | --- | --- | --- | --- | --- | --- | --- | --- | --- | --- | --- | --- | --- | --- | --- | --- | --- | --- |
| Age (years old) | Sex (0=female, 1=male) | Histology (1=AA, 2=AO, 3=GBM, 4=DMG, 5=mixed glioma) | WHO grade | Main location | | | | | No. of glioma (0=no, 1=yes) | Extent of resection (0=PR, 1=STR, 2=NTR, 3=GTR) | Extent of resection (0=PR+STR, 1=GTR+NTR) | IDH mutation (0=no, 1=yes) | MGMT methylation (0=no, 1=yes) | 1p19q deletion (0=no, 1=yes) | Ki-67 | Epilepsy before surgery (0=no, 1=yes) | Preoperative PNI | Status (0=alive, 1=death) | OS (months) |
|  |  |  |  | frontal (0=no, 1=yes) | parietal (0=no, 1=yes) | occipital (0=no, 1=yes) | temporal (0=no, 1=yes) | insular (0=no, 1=yes) |  |  |  |  |  |  |  |  |  |  |  |
| 29 | 0 | 3 | 4 | 1 | 0 | 0 | 0 | 0 | 0 | 2 | 1 | 1 | 1 | 0 | 15 | 1 | 44.70 | 0 | 81 |
| 27 | 0 | 3 | 4 | 1 | 0 | 0 | 0 | 0 | 0 | 3 | 1 | 1 | 1 | 0 | 30 | 1 | 50.10 | 0 | 77 |
| 65 | 1 | 3 | 4 | 0 | 1 | 1 | 1 | 0 | 0 | 2 | 1 | 0 | 1 | 0 | 20 | 0 | 48.45 | 1 | 23 |
| 54 | 1 | 4 | 3 | 1 | 1 | 0 | 1 | 0 | 0 | 2 | 1 | 1 | 1 | 0 | 5 | 0 | 59.85 | 0 | 73 |
| 47 | 1 | 3 | 4 | 1 | 0 | 0 | 0 | 0 | 0 | 3 | 1 | 0 | 0 | 0 | 65 | 1 | 58.75 | 1 | 7 |
| 67 | 0 | 2 | 3 | 1 | 0 | 0 | 0 | 0 | 0 | 1 | 0 | 1 | 1 | 1 | 30 | 0 | 52.10 | 1 | 24 |
| 39 | 0 | 3 | 4 | 1 | 1 | 1 | 0 | 0 | 1 | 3 | 1 | 0 | 0 | 0 | 15 | 0 | 46.35 | 1 | 6 |
| 47 | 0 | 3 | 4 | 1 | 0 | 0 | 0 | 0 | 0 | 3 | 1 | 1 | 1 | 0 | 40 | 0 | 42.55 | 1 | 28 |
| 44 | 0 | 1 | 3 | 1 | 0 | 0 | 0 | 0 | 0 | 3 | 1 | 1 | 1 | 0 | 5 | 0 | 51.60 | 1 | 59 |
| 58 | 0 | 3 | 4 | 1 | 1 | 1 | 0 | 0 | 1 | 2 | 1 | 0 | 0 | 0 | 10 | 0 | 59.05 | 1 | 3 |
| 30 | 0 | 1 | 3 | 1 | 0 | 0 | 0 | 0 | 0 | 0 | 0 | 1 | 0 | 0 | 10 | 0 | 63.65 | 1 | 12 |
| 68 | 1 | 1 | 3 | 0 | 0 | 0 | 1 | 0 | 0 | 3 | 1 | 1 | 1 | 0 | 5 | 1 | 53.25 | 1 | 21 |
| 63 | 0 | 3 | 4 | 1 | 0 | 0 | 0 | 0 | 0 | 3 | 1 | 0 | 0 | 0 | 30 | 0 | 47.25 | 1 | 7 |
| 42 | 1 | 1 | 3 | 1 | 0 | 0 | 0 | 0 | 0 | 3 | 1 | 1 | 1 | 0 | 40 | 1 | 57.05 | 1 | 32 |
| 49 | 0 | 3 | 4 | 1 | 0 | 0 | 0 | 0 | 0 | 3 | 1 | 0 | 0 | 0 | 30 | 0 | 39.25 | 1 | 12 |
| 34 | 1 | 1 | 3 | 1 | 0 | 0 | 0 | 0 | 0 | 3 | 1 | 1 | 1 | 0 | 8 | 1 | 57.95 | 0 | 66 |
| 50 | 0 | 1 | 3 | 0 | 0 | 1 | 1 | 0 | 0 | 1 | 0 | 1 | 1 | 0 | 15 | 0 | 46.30 | 1 | 35 |
| 35 | 0 | 2 | 3 | 1 | 0 | 0 | 1 | 0 | 0 | 2 | 1 | 1 | 0 | 1 | 15 | 0 | 50.40 | 0 | 66 |
| 60 | 1 | 2 | 3 | 1 | 0 | 0 | 0 | 0 | 0 | 3 | 1 | 1 | 1 | 1 | 8 | 1 | 54.65 | 0 | 65 |
| 38 | 1 | 1 | 3 | 0 | 1 | 0 | 0 | 0 | 0 | 3 | 1 | 1 | 0 | 0 | 5 | 1 | 49.00 | 0 | 65 |
| 73 | 1 | 3 | 4 | 1 | 0 | 0 | 0 | 0 | 1 | 1 | 0 | 0 | 0 | 0 | 30 | 1 | 49.65 | 1 | 8 |
| 18 | 0 | 3 | 4 | 0 | 1 | 0 | 1 | 0 | 0 | 3 | 1 | 0 | 0 | 0 | 30 | 0 | 54.00 | 0 | 64 |
| 51 | 0 | 1 | 3 | 1 | 0 | 0 | 0 | 0 | 0 | 2 | 1 | 1 | 1 | 0 | 5 | 0 | 53.70 | 0 | 63 |
| 51 | 1 | 3 | 4 | 1 | 0 | 0 | 1 | 0 | 1 | 3 | 1 | 0 | 0 | 0 | 50 | 0 | 47.50 | 1 | 12 |
| 67 | 1 | 4 | 3 | 0 | 1 | 0 | 0 | 0 | 0 | 3 | 1 | 1 | 0 | 0 | 70 | 0 | 46.80 | 1 | 15 |
| 35 | 1 | 2 | 3 | 1 | 0 | 0 | 1 | 0 | 0 | 2 | 1 | 1 | 1 | 1 | 20 | 1 | 50.30 | 0 | 58 |
| 25 | 0 | 3 | 4 | 0 | 0 | 0 | 0 | 0 | 0 | 3 | 1 | 1 | 1 | 0 | 15 | 0 | 59.15 | 0 | 57 |
| 35 | 0 | 3 | 4 | 0 | 0 | 0 | 0 | 0 | 0 | 3 | 1 | 0 | 1 | 0 | 40 | 0 | 32.20 | 1 | 32 |
| 59 | 1 | 3 | 4 | 1 | 0 | 0 | 0 | 0 | 0 | 3 | 1 | 1 | 1 | 0 | 40 | 1 | 52.35 | 0 | 54 |
| 73 | 0 | 3 | 4 | 1 | 0 | 0 | 0 | 0 | 0 | 1 | 0 | 0 | 1 | 0 | 15 | 0 | 46.75 | 1 | 12 |
| 59 | 1 | 3 | 4 | 0 | 1 | 1 | 1 | 0 | 0 | 3 | 1 | 0 | 1 | 0 | 20 | 0 | 53.00 | 0 | 51 |
| 53 | 1 | 1 | 3 | 0 | 0 | 0 | 1 | 0 | 0 | 0 | 0 | 1 | 0 | 0 | 10 | 0 | 55.40 | 1 | 6 |
| 39 | 0 | 3 | 4 | 1 | 0 | 0 | 0 | 0 | 0 | 1 | 0 | 0 | 0 | 0 | 40 | 0 | 64.30 | 1 | 14 |
| 47 | 1 | 2 | 3 | 1 | 0 | 0 | 0 | 0 | 0 | 1 | 0 | 1 | 1 | 1 | 10 | 1 | 51.10 | 1 | 38 |
| 56 | 1 | 1 | 3 | 1 | 0 | 0 | 0 | 0 | 0 | 3 | 1 | 1 | 0 | 0 | 15 | 0 | 44.45 | 1 | 20 |
| 54 | 1 | 3 | 4 | 1 | 0 | 0 | 0 | 0 | 0 | 3 | 1 | 0 | 1 | 0 | 20 | 0 | 45.90 | 0 | 48 |
| 66 | 1 | 3 | 4 | 0 | 0 | 0 | 1 | 0 | 1 | 3 | 1 | 0 | 0 | 0 | 15 | 1 | 42.70 | 1 | 10 |
| 53 | 1 | 1 | 3 | 0 | 1 | 0 | 0 | 0 | 0 | 3 | 1 | 1 | 1 | 0 | 5 | 0 | 59.90 | 0 | 46 |
| 79 | 1 | 3 | 4 | 1 | 1 | 0 | 0 | 0 | 0 | 3 | 1 | 0 | 0 | 0 | 30 | 0 | 38.80 | 1 | 14 |
| 51 | 1 | 3 | 4 | 1 | 0 | 0 | 0 | 0 | 0 | 2 | 1 | 0 | 1 | 0 | 10 | 0 | 53.35 | 1 | 22 |
| 49 | 1 | 2 | 3 | 1 | 0 | 0 | 0 | 0 | 0 | 2 | 1 | 1 | 0 | 1 | 5 | 1 | 47.55 | 0 | 45 |
| 48 | 1 | 1 | 3 | 1 | 0 | 0 | 0 | 0 | 0 | 3 | 1 | 1 | 1 | 0 | 5 | 1 | 45.50 | 0 | 43 |
| 34 | 0 | 1 | 3 | 0 | 0 | 0 | 1 | 1 | 0 | 2 | 1 | 1 | 0 | 0 | 5 | 1 | 46.35 | 0 | 42 |
| 52 | 0 | 3 | 4 | 1 | 0 | 0 | 1 | 1 | 0 | 3 | 1 | 0 | 0 | 0 | 30 | 0 | 57.05 | 1 | 16 |
| 36 | 1 | 1 | 3 | 0 | 0 | 0 | 1 | 0 | 0 | 3 | 1 | 0 | 1 | 0 | 10 | 0 | 67.05 | 0 | 42 |
| 47 | 1 | 2 | 3 | 1 | 0 | 0 | 0 | 0 | 0 | 3 | 1 | 1 | 1 | 1 | 10 | 1 | 50.30 | 0 | 41 |
| 51 | 1 | 3 | 4 | 0 | 1 | 1 | 0 | 0 | 0 | 2 | 1 | 0 | 0 | 0 | 20 | 0 | 48.95 | 1 | 14 |
| 61 | 1 | 3 | 4 | 0 | 0 | 0 | 1 | 1 | 0 | 2 | 1 | 0 | 0 | 0 | 20 | 1 | 52.50 | 1 | 14 |
| 46 | 1 | 3 | 4 | 1 | 0 | 0 | 0 | 0 | 0 | 2 | 1 | 0 | 0 | 0 | 20 | 0 | 52.45 | 1 | 17 |
| 71 | 1 | 2 | 3 | 1 | 0 | 0 | 0 | 0 | 0 | 3 | 1 | 1 | 0 | 1 | 10 | 0 | 45.60 | 1 | 21 |
| 46 | 0 | 1 | 3 | 1 | 0 | 0 | 0 | 0 | 0 | 3 | 1 | 1 | 1 | 0 | 8 | 0 | 47.25 | 0 | 37 |
| 59 | 1 | 3 | 4 | 0 | 1 | 1 | 0 | 0 | 0 | 3 | 1 | 1 | 1 | 0 | 30 | 0 | 44.35 | 1 | 35 |
| 60 | 0 | 3 | 4 | 1 | 1 | 0 | 0 | 0 | 0 | 3 | 1 | 0 | 1 | 0 | 20 | 0 | 48.90 | 1 | 21 |
| 53 | 1 | 3 | 4 | 1 | 1 | 0 | 0 | 0 | 0 | 3 | 1 | 1 | 1 | 0 | 15 | 0 | 55.15 | 1 | 34 |
| 45 | 0 | 3 | 4 | 1 | 0 | 0 | 0 | 0 | 0 | 3 | 1 | 1 | 1 | 0 | 15 | 0 | 50.20 | 0 | 33 |
| 45 | 1 | 3 | 4 | 0 | 1 | 0 | 1 | 0 | 0 | 3 | 1 | 1 | 1 | 0 | 15 | 0 | 44.40 | 0 | 33 |
| 60 | 1 | 1 | 3 | 0 | 0 | 1 | 0 | 0 | 0 | 3 | 1 | 0 | 1 | 0 | 15 | 0 | 50.85 | 1 | 22 |
| 65 | 1 | 3 | 4 | 0 | 0 | 1 | 0 | 0 | 0 | 1 | 0 | 0 | 0 | 0 | 40 | 0 | 50.05 | 1 | 3 |
| 59 | 0 | 3 | 4 | 0 | 1 | 0 | 0 | 0 | 0 | 3 | 1 | 0 | 0 | 0 | 30 | 0 | 48.80 | 1 | 17 |
| 69 | 0 | 3 | 4 | 0 | 1 | 0 | 0 | 0 | 0 | 3 | 1 | 0 | 0 | 0 | 10 | 0 | 49.30 | 0 | 31 |
| 66 | 1 | 3 | 4 | 1 | 0 | 0 | 0 | 0 | 0 | 1 | 0 | 0 | 0 | 0 | 30 | 0 | 53.00 | 1 | 6 |
| 44 | 0 | 1 | 3 | 0 | 0 | 0 | 0 | 0 | 0 | 3 | 1 | 1 | 1 | 0 | 10 | 0 | 50.50 | 0 | 30 |
| 57 | 0 | 3 | 4 | 0 | 1 | 0 | 0 | 0 | 0 | 3 | 1 | 0 | 0 | 0 | 20 | 1 | 50.65 | 1 | 7 |
| 54 | 1 | 3 | 4 | 1 | 1 | 0 | 0 | 0 | 0 | 3 | 1 | 0 | 0 | 0 | 20 | 0 | 42.25 | 1 | 8 |
| 64 | 0 | 3 | 4 | 1 | 0 | 0 | 0 | 0 | 0 | 3 | 1 | 0 | 1 | 0 | 70 | 0 | 58.95 | 0 | 28 |
| 52 | 0 | 3 | 4 | 0 | 0 | 0 | 1 | 0 | 0 | 3 | 1 | 0 | 0 | 0 | 20 | 0 | 54.50 | 1 | 8 |
| 43 | 0 | 1 | 3 | 1 | 0 | 0 | 0 | 0 | 0 | 3 | 1 | 1 | 0 | 0 | 6 | 1 | 60.45 | 0 | 28 |
| 31 | 0 | 3 | 4 | 0 | 0 | 0 | 0 | 0 | 0 | 3 | 1 | 0 | 0 | 0 | 20 | 0 | 47.00 | 1 | 14 |
| 35 | 1 | 3 | 4 | 0 | 0 | 0 | 1 | 0 | 0 | 3 | 1 | 0 | 0 | 0 | 40 | 0 | 56.20 | 1 | 24 |
| 23 | 1 | 3 | 4 | 0 | 1 | 1 | 0 | 0 | 0 | 2 | 1 | 0 | 1 | 0 | 25 | 0 | 50.80 | 1 | 22 |
| 32 | 0 | 3 | 4 | 1 | 0 | 0 | 1 | 0 | 0 | 3 | 1 | 0 | 1 | 0 | 70 | 0 | 39.00 | 1 | 15 |
| 44 | 0 | 3 | 4 | 0 | 1 | 0 | 0 | 0 | 0 | 3 | 1 | 0 | 0 | 0 | 40 | 0 | 57.05 | 1 | 11 |
| 49 | 1 | 2 | 3 | 0 | 0 | 0 | 1 | 0 | 0 | 3 | 1 | 1 | 1 | 1 | 10 | 0 | 46.85 | 1 | 22 |
| 60 | 1 | 3 | 4 | 0 | 0 | 0 | 0 | 0 | 0 | 3 | 1 | 0 | 0 | 0 | 20 | 0 | 56.05 | 1 | 12 |
| 31 | 1 | 3 | 4 | 1 | 0 | 0 | 0 | 0 | 0 | 3 | 1 | 0 | 0 | 0 | 20 | 0 | 52.50 | 0 | 22 |
| 55 | 1 | 2 | 3 | 1 | 0 | 0 | 0 | 0 | 0 | 3 | 1 | 1 | 1 | 1 | 40 | 0 | 43.40 | 0 | 22 |
| 51 | 1 | 2 | 3 | 1 | 0 | 0 | 0 | 0 | 0 | 3 | 1 | 1 | 1 | 1 | 40 | 0 | 49.25 | 0 | 22 |
| 50 | 0 | 1 | 3 | 1 | 0 | 0 | 0 | 0 | 0 | 3 | 1 | 1 | 1 | 0 | 20 | 0 | 47.80 | 0 | 21 |
| 49 | 1 | 3 | 4 | 1 | 0 | 0 | 0 | 0 | 0 | 3 | 1 | 0 | 0 | 0 | 20 | 0 | 47.25 | 0 | 21 |
| 37 | 0 | 2 | 3 | 1 | 0 | 0 | 0 | 0 | 0 | 3 | 1 | 1 | 0 | 1 | 30 | 1 | 50.50 | 0 | 21 |
| 61 | 0 | 3 | 4 | 1 | 0 | 0 | 0 | 0 | 0 | 1 | 0 | 0 | 1 | 0 | 10 | 0 | 56.90 | 1 | 8 |
| 46 | 0 | 3 | 4 | 1 | 0 | 0 | 0 | 0 | 0 | 3 | 1 | 0 | 0 | 0 | 30 | 0 | 57.25 | 0 | 20 |
| 61 | 0 | 3 | 4 | 1 | 1 | 0 | 0 | 0 | 1 | 3 | 1 | 0 | 0 | 0 | 15 | 0 | 48.15 | 0 | 19 |
| 52 | 1 | 3 | 4 | 0 | 0 | 0 | 1 | 0 | 0 | 3 | 1 | 0 | 1 | 0 | 50 | 0 | 47.20 | 1 | 18 |
| 70 | 0 | 3 | 4 | 0 | 0 | 0 | 0 | 0 | 0 | 3 | 1 | 0 | 0 | 0 | 50 | 0 | 58.40 | 0 | 18 |
| 49 | 0 | 1 | 3 | 1 | 0 | 0 | 0 | 0 | 0 | 3 | 1 | 1 | 0 | 0 | 30 | 0 | 41.55 | 1 | 17 |
| 73 | 0 | 3 | 4 | 1 | 0 | 0 | 0 | 0 | 0 | 3 | 1 | 0 | 0 | 0 | 20 | 0 | 49.80 | 0 | 17 |
| 45 | 1 | 1 | 3 | 1 | 0 | 0 | 0 | 0 | 0 | 3 | 1 | 1 | 0 | 0 | 15 | 0 | 49.70 | 0 | 16 |
| 43 | 1 | 3 | 4 | 0 | 0 | 1 | 0 | 0 | 0 | 3 | 1 | 0 | 0 | 0 | 30 | 0 | 57.65 | 1 | 6 |
| 36 | 0 | 3 | 4 | 1 | 0 | 0 | 0 | 0 | 0 | 3 | 1 | 0 | 0 | 0 | 20 | 0 | 54.30 | 1 | 12 |
| 43 | 0 | 2 | 3 | 1 | 0 | 0 | 0 | 0 | 0 | 3 | 1 | 1 | 0 | 1 | 35 | 0 | 49.95 | 0 | 13 |
| AA: anaplastic astrocytomas, AO: anaplastic oligodendrogliomas, GBM: glioblastoma, GTR: gross total resection, No.: number, NOS: not otherwised speccified, NTR: near total resection, PNI: prognostic nutritional index, PR: partial resection, STR: subtotal resection. | | | | | | | | | | | | | | | | | | | |
|  |  |  |  |  |  |  |  |  |  |  |  |  |  |  |  |  |  |  |  |
